# The safety and efficacy of genicular nerve radiofrequency ablation for pain in inferolateral quadrant of the knee^☆;^

**DOI:** 10.1016/j.inpm.2023.100253

**Published:** 2023-05-31

**Authors:** Weibin Shi, To-Nhu Vu, Thiru Annaswamy, Hong Wu, Bryan Moore, Chad Mears, Allen R. Kunselman

**Affiliations:** a Department of Physical Medicine and Rehabilitation, Penn State Health Milton S Hershey Medical Center/Penn State College of Medicine, Hershey, PA, USA; b Penn State Hershey Rehabilitation Hospital, Hummelstown, PA, USA; c Department of Anesthesiology & Perioperative Medicine, Penn State Health Milton S Hershey Medical Center/Penn State College of Medicine, Hershey, PA, USA; d Department of Physical Medicine and Rehabilitation, RUSH Medical College, Chicago, IL, USA; e Department of Public Health Sciences, Penn State Hershey College of Medicine, Hershey, PA, USA

**Keywords:** Knee pain, Genicular nerve, Recurrent fibular nerve, Radiofrequency ablation, Patient safety

## Abstract

**Background:**

Genicular nerve radiofrequency ablation (RFA) is an effective procedure to alleviate knee pain. This procedure has been proven to be safe, except in the inferior lateral quadrant (ILQ) due to its innervation being near the common fibular nerve (CFN). Given the complexity of this approach, pain physicians do not routinely perform RFA in the ILQ, leading to inadequate pain relief in this region.

**Methods:**

This is a retrospective study of 54 patients who had undergone genicular nerve RFA. Thirty patients had genicular nerve RFA of the knee joint including the ILQ innervated by the inferolateral genicular and recurrent fibular nerves, while 24 patients had RFA of the knee joint without involvement of the ILQ. We compared the outcomes (pain relief, function, and complications) in the patients with and without ILQ RFA at 3 months and 6 months after RFA.

**Results:**

There was no significant difference in initial pain and functional level before RFA between these two groups. After RFA, the two groups had comparable pain relief at 3 months (p ​= ​0.06) and 6 months (p ​= ​0.20), and similar functionality at 3 months (p ​= ​0.29) and 6 months (p ​= ​0.12). There were no reported complications after RFA with or without ILQ RFA.

**Conclusions:**

RFA of the innervation to the ILQ of the knee is as safe and effective as all other anterior quadrants.

Since Choi et al. first introduced radiofrequency ablation (RFA) as a technique to alleviate chronic knee pain secondary to osteoarthritis (OA) in 2011 [1], genicular nerve RFA has emerged as a popular procedure to treat knee pain [2,3]. Due to the complex innervation of the knee joint, RFA for knee joint pain can be challenging, especially for pain localized at the inferior lateral quadrant (ILQ) of the anterior aspect of the knee. The recurrent fibular nerve, the main innervating nerve of the ILQ of the knee, was often not targeted due to its close proximity to the common fibular nerve (CFN).

The recurrent fibular nerve (RFN) branches from the common fibular nerve (CFN), then courses anterosuperiorly around the neck of fibula. Under Gerdy's tubercle, the RFN terminates as 1 to 3 articular branches innervating the inferior part of the quadrant [4,5], consistently the patellar tendon and/or the infrapatellar fat pad [6].

The inferior lateral genicular nerve is found underneath the lateral collateral ligament of the knee, lateral to the lateral meniscus or tibial plateau, supplying the inferolateral and anterior aspects of the knee joint [4,7,8].

Based on the cadaveric dissection studies performed by Tran et al. [4] and cadaveric dye study by Fonkoue et al. [5], a safe approach to the RFN has been proposed [9]. However, no clinical studies have been performed to confirm the safety and efficacy of RFA in the ILQ in comparison to the other 3 common quadrants.

Our objectives were to demonstrate our approaches and evaluate the outcomes of ILQ RFA in comparison to that of RFA of the innervation to the other 3 common quadrants.

## 1 Methods

Permission to conduct this study was granted by the Institutional Review Board at our institute STUDY (00019923). All procedures and follow-up visits were performed between September 2019 and August 2022. A total of 65 patients underwent knee genicular nerve radiofrequency ablation (GNRFA) during this time and were screened.

Inclusion criteria were 1) ages between 18 and 90 years; 2) greater than six months of knee pain; 3) failure of conventional therapy, including oral medications, physical therapy, and intra-articular injection therapy, who were unsuitable surgical candidates because of medical comorbidities and/or a high body mass index (BMI), or wanted to avoid surgery; 4) history of knee surgeries, including total knee arthroplasty with or without revision, but were not candidates for further knee surgery.

Exclusion criteria were 1) contraindications to genicular nerve block or genicular nerve RFA (active infection, bleeding disorders, current anticoagulant or antiplatelet medication use that cannot be safely held, allergy to medications, pregnancy, or use of a pacemaker); 2) clinically significant cognitive deficit, unstable medical or psychiatric illness; 3) failure in providing follow-up information at the six-month duration of the study.

Fifty-four patients met inclusion criteria. Prior to GNRFA, patients were asked to report their pain on a numerical rating scale (NRS) and patient-specific functional scale (PSFS) [[10], [11], [12]], between 0 and 10. Three major functions were evaluated using PSFS, including standing, walking, and stair negotiation.

Diagnostic genicular nerve blocks were performed by the same interventional pain physician who performed the GNRFA procedures in this study. No superficial local anesthesia was used for diagnostic blocks. The needle/probe placement follows as described in literature [2,5,13,14].

**Fig. 1:**
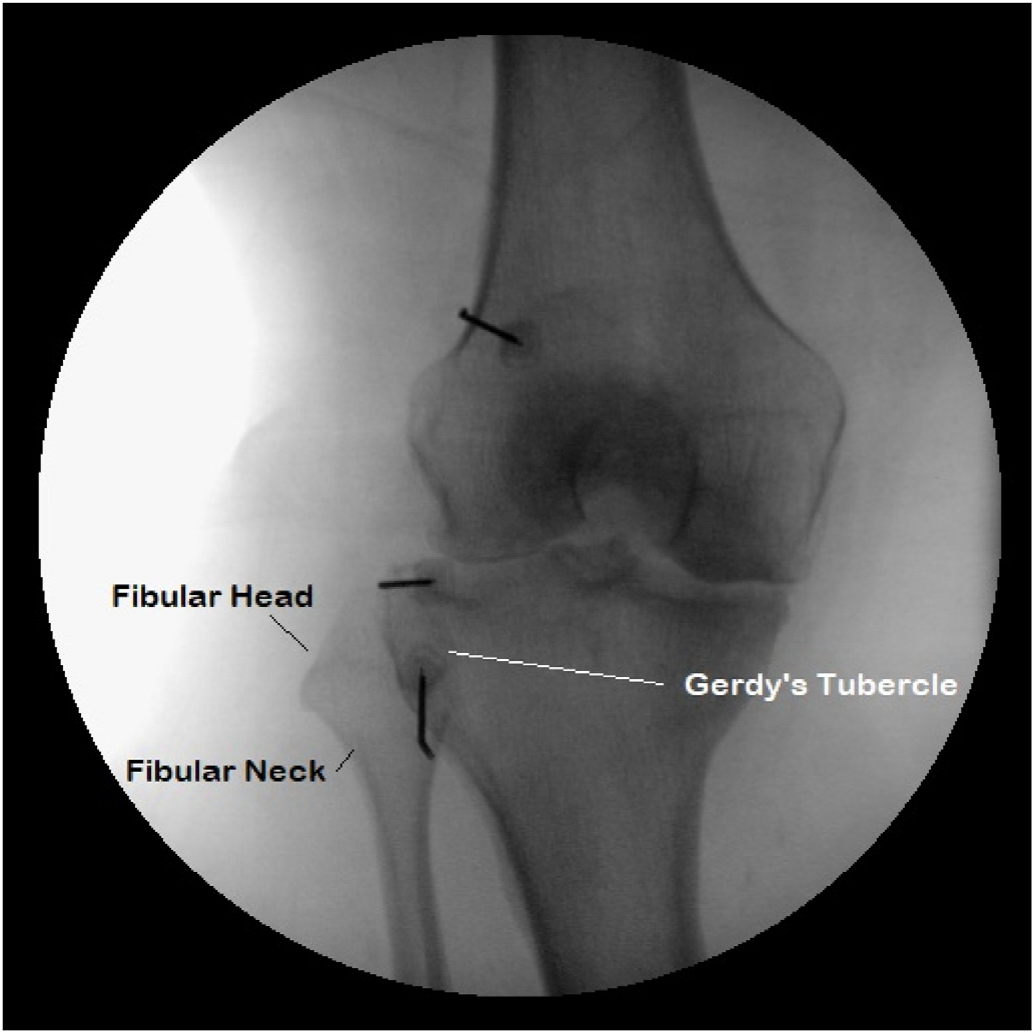
On anterior-posterior view, the inferior RF cannula tip, targeting the recurrent fibular nerve, should be at the level of the fibular neck, between the tibia and fibula. The superior RF cannula tip targets the superolateral genicular nerve and the middle RF cannula tip targets the inferolateral genicular nerve.

**Fig. 2:**
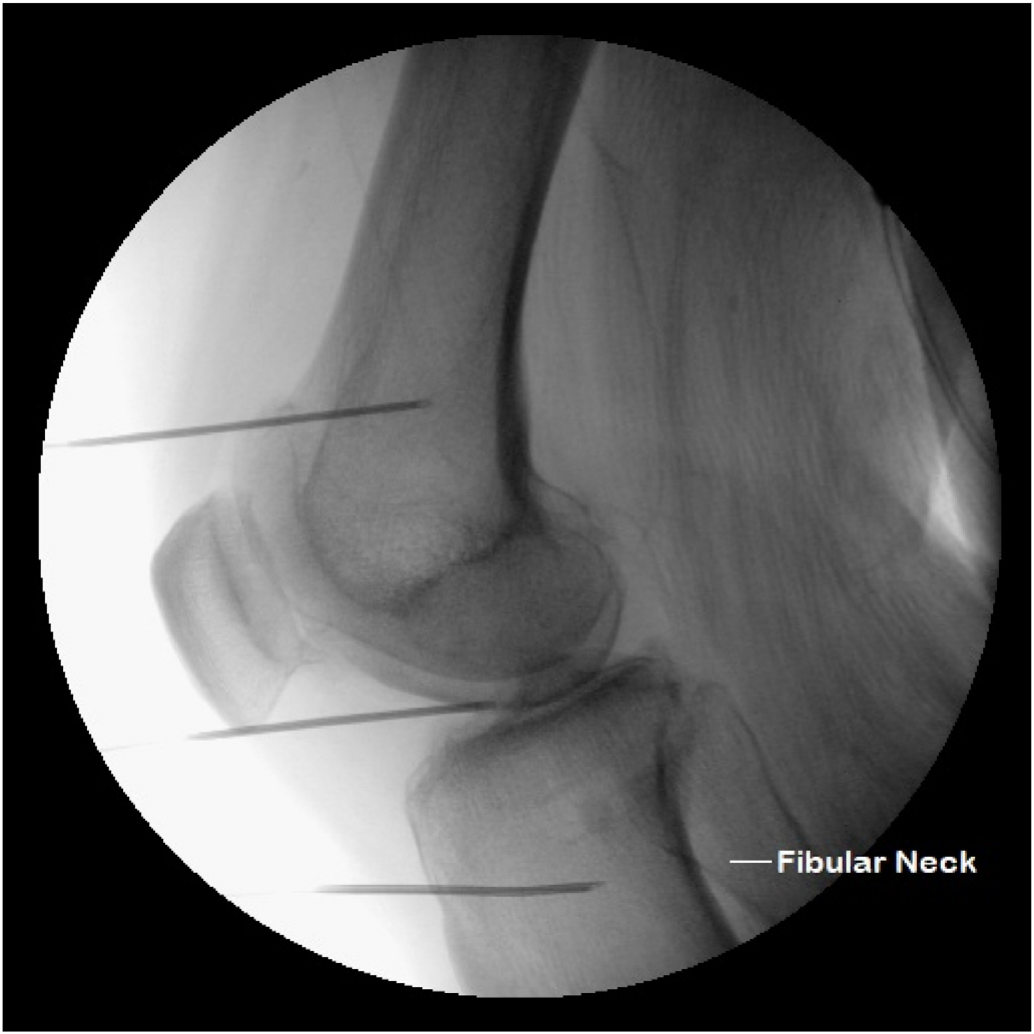
On lateral view, the inferior RF cannula tip, targeting the recurrent fibular nerve, should be approximately 0.5 ​cm anterior to the fibular neck level. The superior RF cannula tip targets the superolateral genicular nerve and the middle RF cannula tip targets the inferolateral genicular nerve.

GNRFA was performed at an outpatient surgery center. All patients received monitored anesthesia care prior to the procedure. The target for needle placement was as described previously [14] except for the RFN. The entry point for the RFN is about 1–3 ​cm below Gerdy's tubercle depending on anatomical variation. The needle was inserted towards the medial aspect of the fibular neck but stopped at or above the neck of the fibular head, laterally between the tibia and fibula, and did not pass the longitudinal midline of the fibular head from the lateral aspect (Fig. 1, Fig. 4). From a lateral view, the needle tip should be anterior to the fibular neck (Fig. 2). Normal saline was injected to raise the skin to create about a 1 ​cm separation from the probe in order to prevent damage of the epidermis if necessary.

Following RFA, we routinely followed patients at 2 weeks, 3 months, and 6 months for evaluation of pain relief (NRS) and functional improvement (PSFS). The NRS and PSFS numbers reported before and after ablation were used to calculate a percentage of pain relief. Two patients underwent an additional RFA, and one patient underwent a total knee replacement 6 months after RFA due to unresolved pain and impaired function. We included these 3 patients and documented the pain relief and functional improvement as 0 ​at 6 months. Patients with incomplete follow-up data were not included.

## 2 Statistical analysis

A Wilcoxon rank sum test was performed to analyze if the mean pain relief and function recovery after RFA in patients with inferolateral quadrant (ILQ) pain and ILQ RFA differs from patients without ILQ pain and ILQ RFA. Alpha levels were set at 0.05.

A two-week follow-up appointment was scheduled for postprocedural checks. Given ongoing recovery and soreness at the injection site, patient efficacy data was not analyzed at the two-week timeframe to avoid confounding variables.

## 3 Results

We included 54 patients who met the inclusion and exclusion criteria: 17 males (31.48%), 37 females (68.52%), average age 52.22 years, average BMI 33.81 ​kg/m^2^, and 13 (24.07%) with diabetes mellitus. Thirty (55.56%) patients underwent RFA in the ILQ area, and 24 (44.44%) with no ILQ nerve involved. Fifteen patients (27.78%) were status post total knee arthroplasty; 8 underwent RFA without ILQ and 7 underwent RFA with ILQ. One patient with a history of partial knee arthroplasty underwent RFA with ILQ (Table 1). The prior pain level and functional status are similar in these two groups: pain (p 0.43) and PSFS (p 0.77) (Table 2).

**Table 1:** Demographic and clinical summary.

	%	Mean ​± ​SD
**Gender**	Male: 17/54, 31.48%	
	Female: 37/54, 68.52%	
**Age**		55.22 ​± ​15.17
**BMI (kg/m**^**2**^**)**		33.81 ​± ​7.59
**Status Post Knee Arthroplasty**	TKA: 15/54, 27.78%	
	PKA: 1/54, 1.85%	
**Diabetes Mellitus**	13/54, 24.07%	
**Inclusion of ILQ**	30/54, 55.56%	

BMI: body mass index; TKA: total knee arthroplasty; PKA: partial knee arthroplasty; SD: standard deviation; ILQ: inferolateral quadrant.

**Table 2:** Wilcoxon Rank-Sum test – ILQ inclusion as binary.

	ILQ	Median (25th percentile, 75th percentile)	Hodges-Lehmann Location Shift Estimate (95% Confidence Interval)	p-value
Pain Pre-RFA	Y	8.0 (7.0, 8.0)	0.0 (−1.0, 1.0)	0.43
	N	8.5 (7.0, 9.0)		
PSFS Pre-RFA	Y	3.0 (3.0, 4.0)	0.0 (−1.0, 0)	0.77
	N	4.0 (3.0, 4.0)		
Pain 3 months (%)	Y	55.0 (50.0, 75.0)	−15.0 (−25.0, 0)	0.06
	N	75.0 (60.0, 90.0)		
PSFS 3 months (%)	Y	50.0 (28.6, 71.4)	−7.1 (−23.8, 8.9)	0.29
	N	64.6 (40.2, 83.3)		
Pain 6 months (%)	Y	45.0 (0, 60.0)	−10.0 (−25.0, 0)	0.20
	N	55.0 (45.0, 60.0)		
PSFS 6 months (%)	Y	28.6 (0, 50)	−12.6 (−28.6, 0)	0.12
	N	50.0 (28.6, 61.9)		

ILQ: inferolateral quadrant; RFA: radiofrequency ablation; PSFS: patient specific functional scale.

Both groups reported no complications after RFA except minor superficial bruises at the needle entry sites observed at the 2-week follow-up.

The Wilcoxon rank sum test showed no significant difference in pain relief between the groups with and without ILQ RFA at 3-month (p 0.06) and 6-month (p 0.20) follow-ups. Additionally, there was no significant difference in functional improvement at 3-month (p 0.29) and 6-month (p 0.12) follow-ups. (Table 2).

## 4 Discussion

Genicular nerve ablations have been utilized as effective modalities for patients with intractable knee pain refractory to other conservative treatments [[1], [2], [3],^7^]. Most sensory nerve branches of the knee are not near the motor nerves, except for the recurrent fibular nerve. For patients’ safety, providers omit this nerve when planning RFA for knee pain. However, for patients with ILQ knee pain, excluding this nerve supplying will likely lead to inadequate pain relief and functional recovery.

Meticulous anatomical dissection data have provided us with excellent approaches to safely access the innervation of the ILQ [[4], [5], [6]]. The cadaveric study by Fonkoue et al. demonstrated an approach 1 ​cm below Gerdy's tubercle, resulting in 100% accuracy without encroaching on the CFN [5]. This approach was further tested clinically by Sperry et al. [9] We used a similar approach with minor modifications. We modified our approach from a perpendicular to an oblique trajectory (towards the medial cortex of the fibular neck) to ensure longitudinal alignment of the RF cannula along the RFN (Fig. 4) for a more thorough ablation. This approach also avoids inadvertent injury of the anterior tibial artery, the major artery supplying the anterior compartment, by using the medial aspect of fibula as a shield. We elicited 2nd and 3rd extensor digitorum longus (EDL) movement during motor stimulation in one patient (1 out of 30). The reinforces the importance of motor testing in order to avoid inadvertent motor nerve injury.

Orthopedic surgeons are facing the same risk when performing any percutaneous or surgical procedures around the proximal fibula. Gerdy's safe zone (free of common fibular nerve and its branches) was proposed [15], an arc of approximately 100° around Gerdy's tubercle, starting at the fibular head and ending at the tibial crest. The distance between Gerdy's tubercle and the head of the fibula determines the radius of Gerdy's safe zone for the fibular nerve, which is usually about 4.5 ​cm [15]. As pain interventionists, we should be mindful that the injury from RFA is different from that of direct instrumentation by surgeons. A lesion from RFA is about 1 ​cm in diameter and a lesion from cooled RFA can be greater than 1 ​cm in diameter. Thus, an extra >0.5 ​cm space must be left to prevent inadvertent nerve injury.

**Fig. 3:**
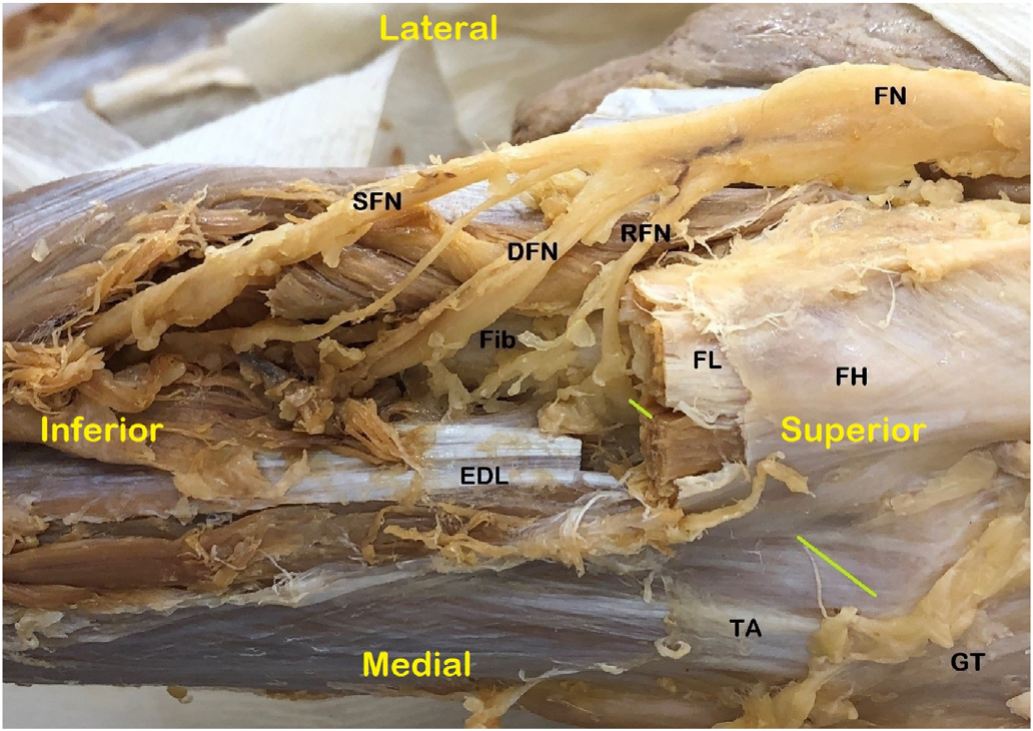
The cadaveric dissection of the recurrent fibular nerve and proposed needle placement. FN: fibular nerve; SFN: superficial fibular nerve; DFN: deep fibular nerve; RFN: recurrent fibular nerve; Fib: fibula; FL: fibularis longus muscle; FH: fibular head; EDL: extensor digitorum longus; TA: tibialis anterior; GT: Gerdy's tubercle; green line: the needle/cannula position.

Our cadaveric dissection shows the proximity of the RFN to the motor branches (Fig. 3). The RFN pierces some muscle fibers in the anterior compartment (tibialis anterior, flexors digitorum longus) before reaching its distribution areas. Targeting the RFN about 1 ​cm away from this bifurcation level will avoid inadvertent ablation of these motor branches. With cooled RFA, these motor branches might be within the radius of the RF lesion. In cases with minor focal muscle fiber twitches, the needle may need to be adjusted. It is still safe to perform RFA if the twitches are focal and only at high motor stimulation levels (>1.5 ​V). These muscle twitch movements are not from stimulation of the major nerve branch. No neurological complications were found in either group of this study.

**Fig. 4:**
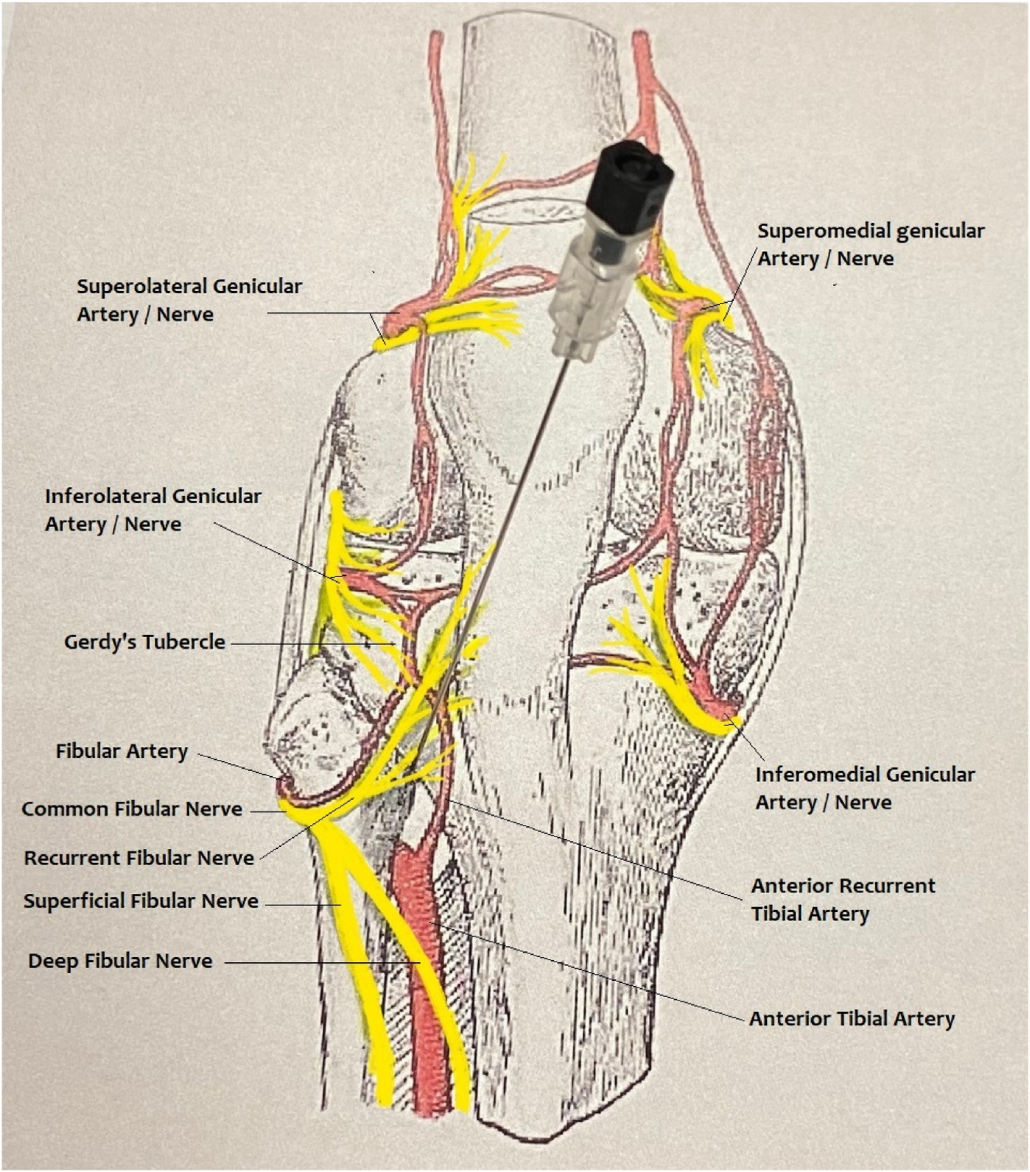
The anatomy sketch illustrates the recurrent fibular nerve in close proximity with the deep fibular nerve and anterior tibial artery. Our proposed needle placement, pictured above, is 1–3 ​cm below Gerdy's tubercle, towards the medial aspect of the fibula at or above the fibular neck. Modified from Gray's Anatomy 1918 (Genicular Arteries).

The risk of vascular injury at the ILQ is similar to that of the other 3 quadrants [16]. Due to the anatomical nature of these nerves being in close proximity to vessels of the same name, RFA can inevitably cause vascular injury in this region. Fortunately, the genicular vascular network offers abundant collateral supply. Symptomatic vascular complications from genicular nerve RFA are rare, but can be serious [17].

While vascular injury is one of our concerns, ironically, genicular artery embolization (GAE) is a novel therapy to treat symptomatic knee osteoarthritis by reducing synovial arterial hypervascularity. A recent GAE study showed that among 40 patients, asymptomatic small bone infarcts were identified in 2 patients on 3-month follow-up MRIs. Since follow-up MRI was not routinely performed in our study, the occurrence of bone infarct or osteonecrosis after RFA was unknown. However, we can extrapolate from the GAE data that RFA likely causes similar complications. This is another reason that an oblique approach was chosen to target the recurrent fibular nerve, as this approach prevents the RF cannula from advancing too far posteriorly to damage the anterior tibial artery and its branch, the anterior tibial recurrent artery (Fig. 4).

Furthermore, it is important to observe precautions for other complications, such as septic arthritis and skin lesions [[18], [19], [20]]. In addition to sterile technique, the best way to avoid septic arthritis is to avoid advancing into the joint space, especially for knee pain after total knee arthroplasty. Periprosthetic joint infection remains one of the most devastating and costly complications after total knee arthroplasty. Encroaching the joint sometimes is inevitable in the superior quadrant locations because the suprapatellar bursa communicates with the knee joint in about 84% adults [21]. We encountered one case of skin burn, similarly reported by McCormick et al. [20], when initially performing GNRFA. Since this complication, we began measuring the distance from the skin to the active tip by using the RF cannula bent tip (roughly 1 ​cm) as a standard gauge. If the GN is within 1 ​cm, we raise the skin by injecting normal saline, which is a common preventive measure used by providers [9]. If anesthetic solution was used instead of normal saline, we ensured it was injected after a motor test. Some practices are still using conventional RFA machines; it is important to keep it in mind that the conventional RF develops heat above the active tip. With these safety measures in place, no skin lesions occurred in patients participating in this study.

In conclusion, while caution should be exercised, RFA of the innervation to the ILQ of the knee is as safe and effective as that of the other 3 anterior quadrants.

## Declaration of competing interest

The authors declare that they have no known competing financial interests or personal relationships that could have appeared to influence the work reported in this paper.
